# Transcriptome analysis revealed key prognostic genes and microRNAs in hepatocellular carcinoma

**DOI:** 10.7717/peerj.8930

**Published:** 2020-04-08

**Authors:** Xi Ma, Lin Zhou, Shusen Zheng

**Affiliations:** 1 Division of Hepatobiliary and Pancreatic Surgery, Department of Surgery, First Affiliated Hospital, School of Medicine, Zhejiang University, Hangzhou, Zhejiang, China; 2 NHC Key Laboratory of Combined Multi-organ Transplantation, Hangzhou, Zhejiang, China; 3 Key Laboratory of the Diagnosis and Treatment of Organ Transplantation, CAMS, Hangzhou, Zhejiang, China; 4 Key Laboratory of Organ Transplantation, Hangzhou, Zhejiang, China

**Keywords:** Transcriptome, Differentially expressed genes, microRNAs, Hepatocellular carcinoma

## Abstract

**Background:**

Hepatocellular carcinoma (HCC) is one of the most common cancers worldwide. However, the molecular mechanisms involved in HCC remain unclear and are in urgent need of elucidation. Therefore, we sought to identify biomarkers in the prognosis of HCC through an integrated bioinformatics analysis.

**Methods:**

Messenger RNA (mRNA) expression profiles were obtained from the Gene Expression Omnibus database and The Cancer Genome Atlas-Liver Hepatocellular Carcinoma (TCGA-LIHC) for the screening of common differentially expressed genes (DEGs). Function and pathway enrichment analysis, protein-protein interaction network construction and key gene identification were performed. The significance of key genes in HCC was validated by overall survival analysis and immunohistochemistry. Meanwhile, based on TCGA data, prognostic microRNAs (miRNAs) were decoded using univariable and multivariable Cox regression analysis, and their target genes were predicted by miRWalk.

**Results:**

Eleven hub genes (upregulated ASPM, AURKA, CCNB2, CDC20, PRC1 and TOP2A and downregulated AOX1, CAT, CYP2E1, CYP3A4 and HP) with the most interactions were considered as potential biomarkers in HCC and confirmed by overall survival analysis. Moreover, AURKA, PRC1, TOP2A, AOX1, CYP2E1, and CYP3A4 were considered candidate liver-biopsy markers for high risk of developing HCC and poor prognosis in HCC. Upregulation of hsa-mir-1269b, hsa-mir-518d, hsa-mir-548aq, hsa-mir-548f-1, and hsa-mir-6728, and downregulation of hsa-mir-139 and hsa-mir-4800 were determined to be risk factors of poor prognosis, and most of these miRNAs have strong potential to help regulate the expression of key genes.

**Conclusions:**

This study undertook the first large-scale integrated bioinformatics analysis of the data from Illumina BeadArray platforms and the TCGA database. With a comprehensive analysis of transcriptional alterations, including mRNAs and miRNAs, in HCC, our study presented candidate biomarkers for the surveillance and prognosis of the disease, and also identified novel therapeutic targets at the molecular and pathway levels.

## Introduction

Liver cancers rank fourth in terms of cancer-related mortality and are the sixth leading cause of new incident cases worldwide (Villanueva, 2019). The 5-year survival rate of liver cancers is 18.1% (Jemal et al., 2017) in the United States, while a worse outcome is reported in China, with a 5-year survival of 12.1% observed (Zheng et al., 2018). In China, approximately 466,100 new cases of liver cancers and approximately 422,100 liver cancer deaths occurred in 2015 (Chen et al., 2016b). Hepatocellular carcinoma (HCC), accounting for the 70–85% of liver cancer cases (Sia et al., 2017), mostly develops in the patients with chronic liver diseases, such as hepatitis B virus or hepatitis C virus (HBV or HCV) infection (Idilman et al., 1998), alcohol abuse (Morgan, Mandayam & Jamal, 2004) or non-alcoholic fatty liver disease (Dyson et al., 2014; Kanwal et al., 2016). Although resection, local ablation, transplantation, transarterial chemoembolization (TACE) and systemic therapies have been applied in the treatment of HCC (Forner, Reig & Bruix, 2018), the survival rate of HCC patients is still low, partly due to the high heterogeneity of HCC (Imamura et al., 2003). Thus, a comprehensive understanding of the transcriptional alterations may contribute to the development of preventive, diagnostic and therapeutic strategies for HCC.

With the advent of next-generation sequencing and microarray technologies at genome level, a large amount of RNA data has been generated and deposited in public databases, such as the Gene Expression Omnibus (GEO) and The Cancer Genome Atlas (TCGA), which enables investigators worldwide to identify differentially expressed genes (DEGs) and associated pathways involved in the onset and development of HCC and other diseases (Clough & Barrett, 2016; Lee, Tan & Chung, 2014). However, because of the heterogeneity of samples and microarray platforms, the results from different single cohort investigations may be inconsistent (Zhao et al., 2018). Therefore, an integrated bioinformatics analysis of multiple independent studies provides more reliable and robust results. Additionally, most analyses are based on the Affymetrix microarray profiles (Li et al., 2017), but considerably less is known about the datasets from Illumina microarray platforms.

Moreover, microRNAs (miRNAs) are short noncoding RNAs and regulate gene expression at the post-transcriptional level. They are involved in the regulation of key biological processes such as cell proliferation, metabolism, differentiation and apoptosis (Sticht et al., 2018). It has been reported that miRNAs contribute to the development of tumors (Iorio & Croce, 2017). However, associations of miRNAs with the dysregulation of gene expression in HCC and the prognosis of HCC patients require further investigation.

In this study, four transcriptome profiles from Illumina microarray platforms and one from TCGA database were collected to systematically identify a set of DEGs. Further analyses on gene ontology (GO), signaling pathways and protein-protein interactions (PPIs) were performed followed by key gene identification, overall survival (OS) analysis and immunohistochemistry validation. Meanwhile, miRNAs functioning as prognostic biomarkers were identified and the potential correlations of miRNA species with that of key genes were investigated.

## Material and Methods

### Profile selection and Data Collection

**Figure 1:**
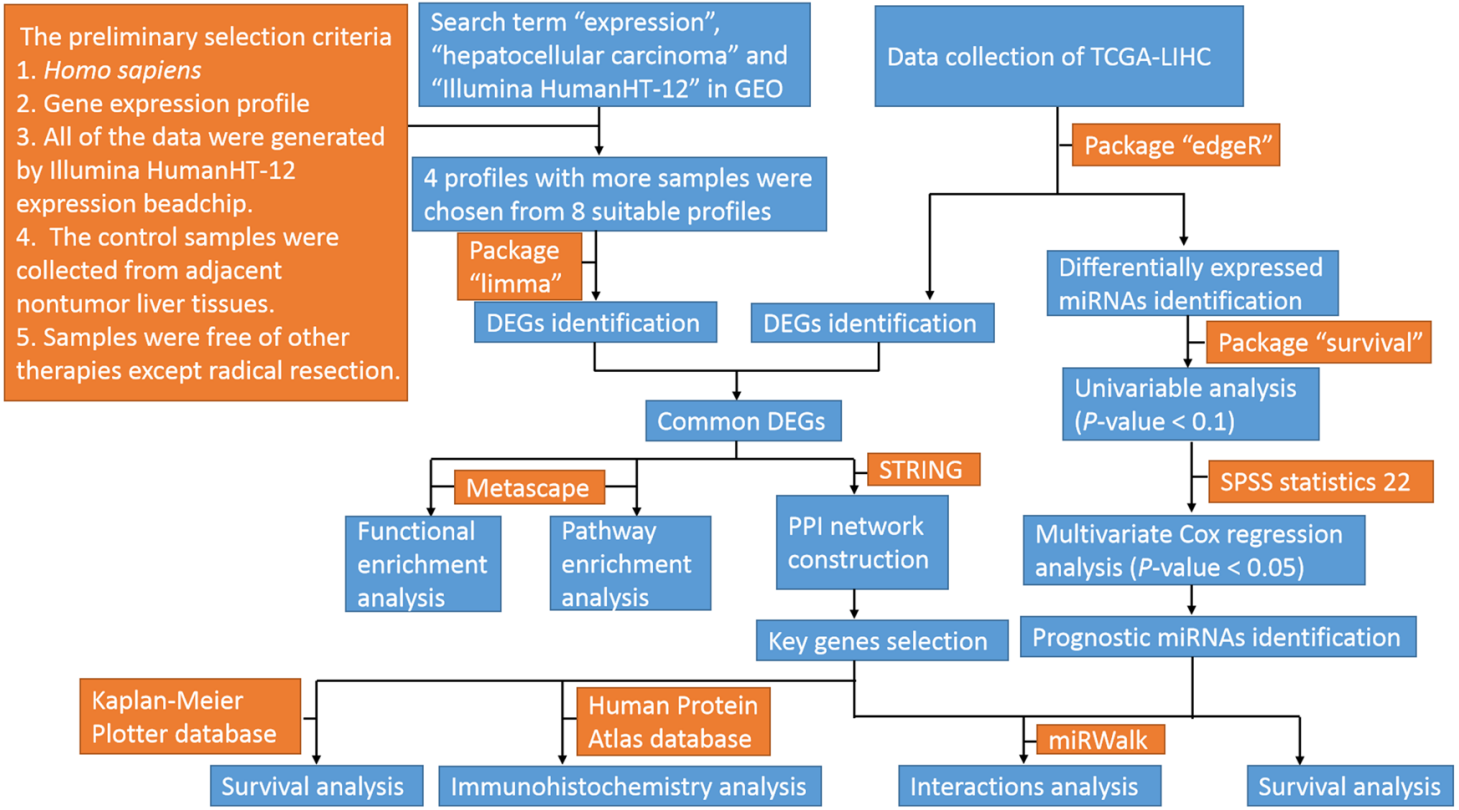
Flow diagram of the study. GEO, gene expression omnibus; TCGA-LIHC, the cancer genome atlas-liver hepatocellular carcinoma; miRNAs, microRNAs; DEGs, differentially expressed genes; PPI, protein–protein interaction.

The profiles in GEO databases were selected according to our selection criteria. The key words used were “expression”, “hepatocellular carcinoma” and “Illumina HumanHT-12”. For the 94 search results, the preliminary selection criteria were as follows: 1. *Homo sapiens*, 2. Gene expression profile; 3. All of the data were generated by an Illumina HumanHT-12 expression beadchip; 4. The control samples were collected from adjacent nontumor liver tissues; and 5. Samples were free of other therapies except radical resection. Among the eight profiles meeting the criteria, four datasets with the most samples were chosen for further analyses. The datasets were GSE36376, GSE39791, GSE57957, and GSE87630. The original Series Matrix Files of these four messenger RNA expression profiles were downloaded from the GEO database (https://www.ncbi.nlm.nih.gov/geo/). The microarray platform of the GSE36376, GSE39791, and GSE57957 datasets was GPL10558 (Illumina HumanHT-12 V4.0 expression beadchip, Illumina Inc.). The GSE87630 dataset was generated by GPL6947 (Illumina HumanHT-12 V3.0 expression beadchip, Illumina Inc.). A total of 415 HCC samples and 334 nontumor samples were included in these four datasets. The mRNA, miRNA and clinical data from TCGA-LIHC (https://cancergenome.nih.gov/) were also downloaded. The flow diagram of the study is shown in Fig. 1.

### Data processing and identification of DEGs

For the mRNA data from GEO, background correction, quantile normalization and batch normalization were performed using R software (version 3.6.1, R Core Team, 2019). Based on the annotation information in the platforms, the probe sets were converted into the corresponding gene symbols. Probe sets without corresponding gene symbols were removed and the expression of genes with more than one probe set was averaged. The bioconductor (http://www.bioconductor.org) package “limma” was employed for DEG screening. *P*-value < 0.05 and —log_2_(Fold Change)—>1 were set as the cut-off criteria for the identification of DEGs in GEO datasets.

For TCGA data, the bioconductor package “edgeR” was applied to calculate expression alterations of genes and miRNAs. The cut-off criteria for differentially expressed genes and miRNAs were consistent with those for GEO data.

The common DEGs identified in both databases were chosen for further analyses. The Venn diagram was generated by FunRich (version 3.1.3, http://www.funrich.org/).

### Functional and signaling pathway enrichment analysis

Metascape (Zhou et al., 2019) (http://metascape.org) was utilized for molecular function (MF), cellular component (CC) and biological processes (BP) enrichment analyses of the common DEGs at the functional level. Pathway enrichment analysis was performed based on three databases in Metascape including KEGG Pathway, Reactome and BioCarta. A *P*-value < 0.05 was defined as significant in both GO and pathway enrichment analyses. The R package “ggplot2″was employed to generate the bubble plots.

### PPI network construction and cluster identification

The PPI network of DEGs was constructed using STRING (version 11.0, http://string-db.org) and a combined score > 0.7 (high confidence) was set as the cut-off criterion. Cytoscape (version 3.7.2, https://cytoscape.org/), an open source platform, was applied to the visualization of molecular interaction networks based on the results from STRING. The Molecular Complex Detection (MCODE) plugin (version 1.5.1) in Cytoscape was used for identifying densely interconnected clusters. The selection criteria were as follows: degree ≥ 2, node score ≥ 0.2, K-core ≥ 2, and max depth = 100.

### Key gene selection and further analyses

The top 11 hub genes in the PPI network with most connections were defined as key genes. The survival analyses of the key genes were performed with the aid of the Kaplan–Meier Plotter database (http://www.kmplot.com). Moreover, the protein expression and distribution in HCC and nontumor samples were displayed with the Human Protein Atlas (HPA) database (Uhlen et al., 2015) (https://www.proteinatlas.org/).

### Prognostic signature of miRNAs and target key genes

The R package “survival” was applied for univariable analysis of differentially expressed miRNAs for overall survival. Then, miRNAs with a *P*-value < 0.1 in univariate analysis were enrolled in multivariable Cox regression analysis using IBM SPSS statistics 22 and a *P*-value < 0.05 was set as the selection criterion. The prognostic model was visualized by a nomogram. Next, Kaplan–Meier plotter analysis was performed to estimate the survival difference between the low-risk and high-risk groups, and 3-year and 5-year overall survival were displayed by ROC curves based on the prognostic model. Moreover, miRWalk (version 3.0, http://mirwalk.umm.uni-heidelberg.de/) was used to determine the overlap between predicted target genes of prognostic miRNAs and key genes identified previously.

## Results

### DEG identification in HCC

**Figure 2:**
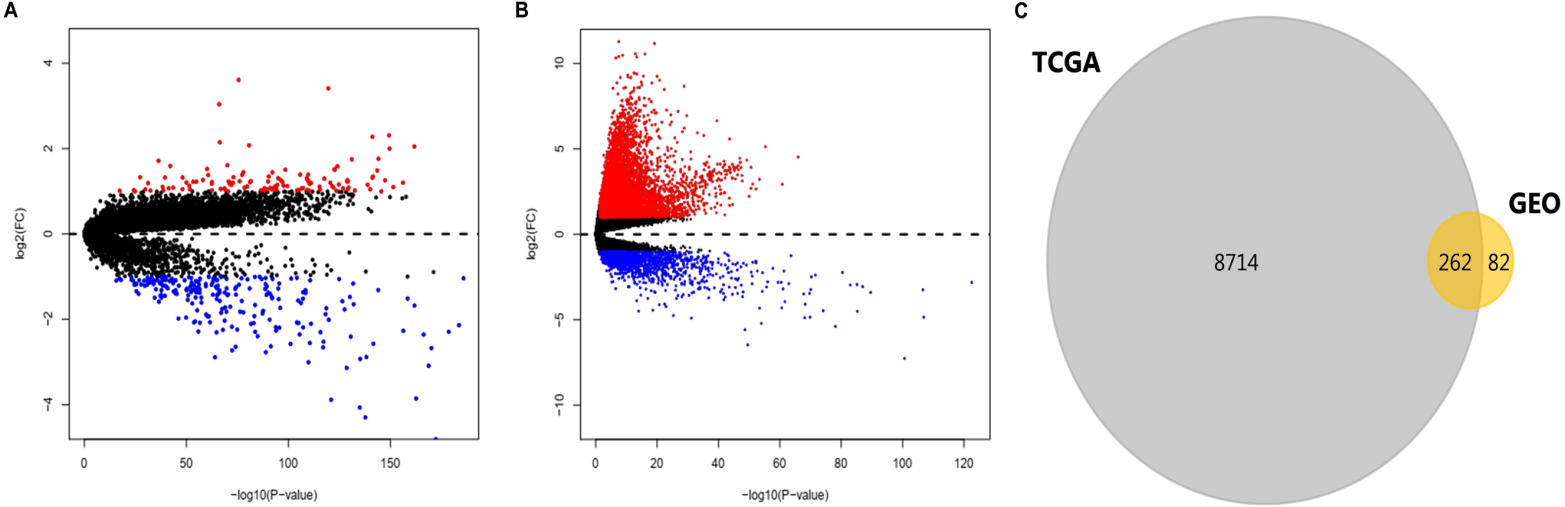
DEGs in HCC identified in four datasets from GEO and one from TCGA. ** DEGs were defined with *P*-value < 0.05 and —log_2_FC— > 1. (A) Volcano plots of genes with expression alteration in HCC samples from GSE36376, GSE39791, GSE57957, and GSE87630. (B) Volcano plots of genes with expression alteration in HCC samples from TCGA database. *X*-axis shows the *P-* value (log-scaled), and *Y*-axis displays the fold change (log-scaled) between HCC samples and non-tumor samples. Each dot represents one gene. The red and green dots represent upregulated and downregulated DEGs, respectively. (C) The common DEGs identified in both databases. DEGs, differentially expressed genes; HCC, hepatocellular carcinoma; GEO, gene expression omnibus; TCGA, the cancer genome atlas; FC, fold change. n.

After standardization of the data in GSE36376, GSE39791, GSE57957, and GSE87630, 344 DEGs (117 upregulated genes and 227 downregulated genes) were identified (Table S1). Meanwhile, 8976 DEGs (7425 upregulated genes and 1551 downregulated genes) were identified in the TCGA dataset (Table S2). The distribution patterns of expressed genes in GEO and TCGA samples are displayed in Figs. 2A and 2B, respectively. Red dots in the Volcano plots represent significantly upregulated genes, while green dots represent significantly downregulated genes. Finally, 81 upregulated genes and 181 downregulated genes (262 DEGs in total) (Fig. 2C, Table 1) identified in both databases were further analyzed.

### GO enrichment analysis of DEGs

**Figure 3:**
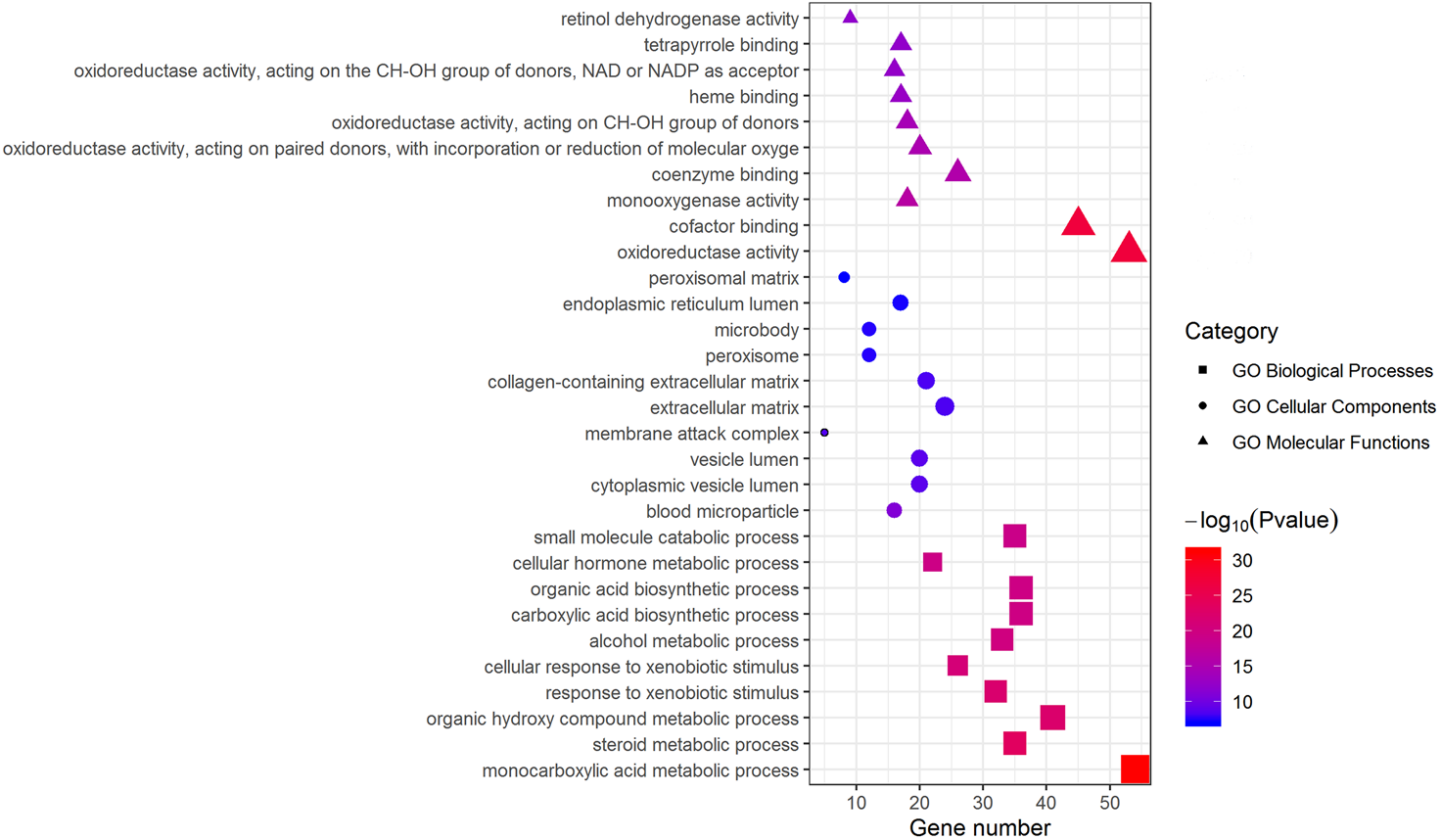
GO enrichment analysis of DEGs. The top 10 (a ranking by *P*-value) significantly enriched GO terms in biological process group, cellular component group and molecular function group were displayed, respectively. Larger size of a symbol represents a higher gene number. GO, gene ontology; DEGs, differentially expressed genes.

Metascape was used for GO biological process, cellular component, and molecular function analyses. The top 10 GO terms with the lowest *P*-value in each group are exhibited in Fig. 3. In terms of molecular functions, the DEGs were significantly enriched in metabolic processes, including monocarboxylic acid, steroid and alcohol metabolic activities, as well as oxidoreductase activity and monooxygenase activity. For the cellular component group, “blood microparticle,” “vesicle lumen” and “extracellular matrix” were the main enriched categories. Specifically, changes in biological processes of the upregulated DEGs significantly focused on chromatid segregation, cell division and nuclear division while metabolic processes accounted for the majority of enriched categories with regard to the downregulated DEGs (Table 2).

### Signaling pathway enrichment analysis of DEGs

**Figure 4:**
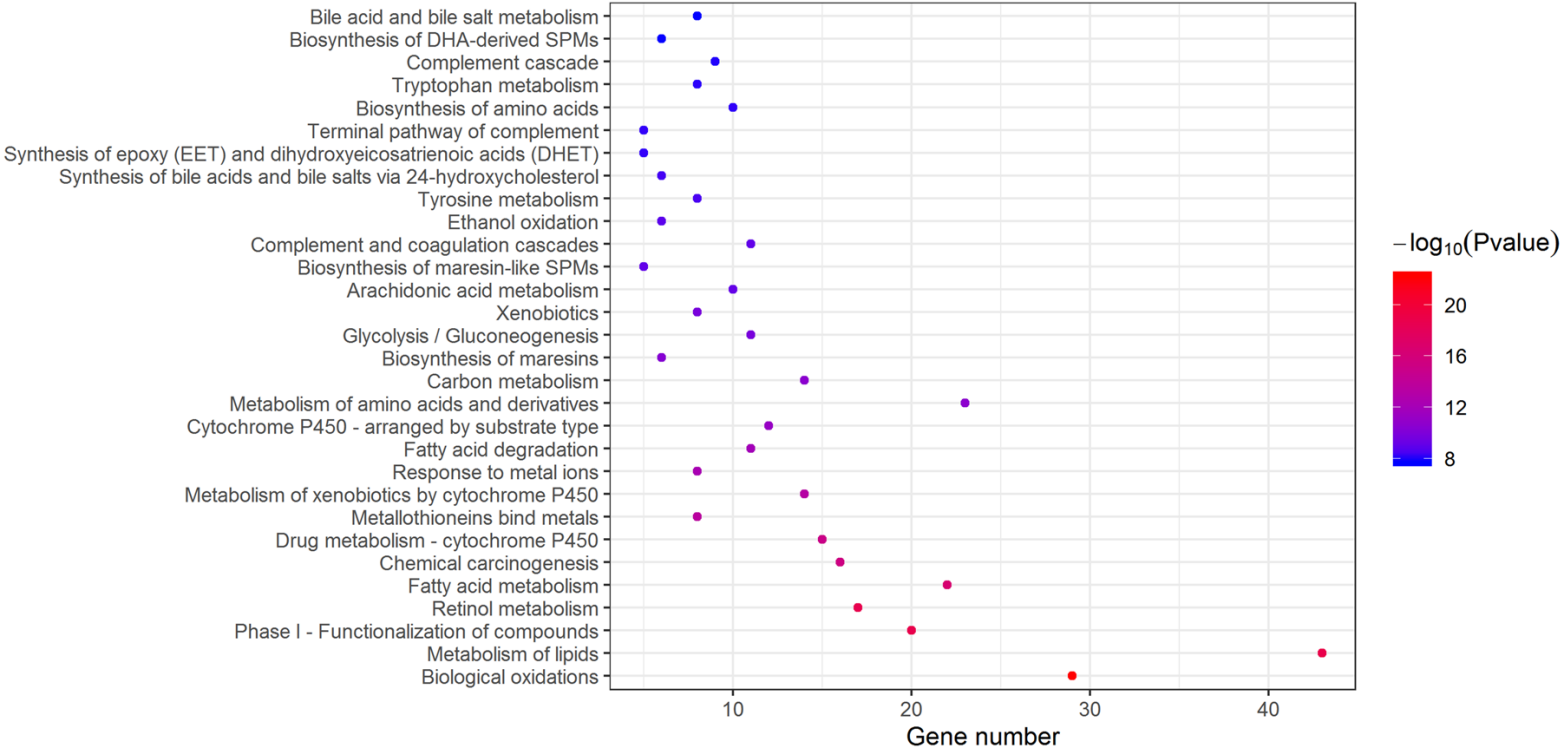
Enriched signaling pathways of DEGs. Based on KEGG pathway, Reactome, BioCarta, 30 enriched pathways with lowest *P*-value were displayed. DEGs, differentially expressed genes.

According to the databases of KEGG Pathway, Reactome and BioCarta gathering in Metascape, biological oxidations and metabolism-related pathways were the main enriched pathways in DEGs (Fig. 4). As shown in Table 3, extracellular matrix organization, cell cycle and collagen formation were significantly enriched in upregulated DEGs. For downregulated DEGs, the top enriched signaling pathways were biological oxidations, retinol metabolism and metabolism of lipids.

**Table 1:** 262 DEGs filtered in HCC samples from GSE36376, GSE39791, GSE57957, GSE87630 and TCGA-LIHC.

**DEGs**	**Gene names**
Upregulated	ACLY ACTG2 AKR1B10 AKR1C3 ASPM AURKA BAIAP2L2 BOP1 BRSK1 CAP2 CCL20 CCNB2 CCT3 CD24 CDC20 CDCA5 CDKN3 CKAP2L CKAP4 CNIH4 COL1A1 COL4A1 COX7B2 DNMT1 EEF1A2 FAM83H FASN GBA GBP2 GLA GPAA1 GPC3 H2AFZ HKDC1 HSPB1 IGF2BP2 IRAK1 IRX3 KIFC1 LAMC1 LAPTM4B LCN2 LOXL4 LYZ MCM2 MCM6 MMP9 MUC13 NCAPG NDUFA4L2 NEU1 NSMCE2 NUSAP1 PAFAH1B3 PEA15 PIK3R2 PITX1 PLOD3 PLVAP PRC1 PTTG1 RAP2A S100A10 S100P SAC3D1 SF3B4 SIPA1L2 SMG5 SNRPB SPINK1 SPP1 SQLE SRXN1 TEAD2 THY1 TKT TMEM106C TMEM45B TOP2A UBD VWF
Downregulated	ACAA1 ACAA2 ACACB ACAD11 ACADS ACOT12 ACSL1 ACSM2A ADAMTSL2 ADH1A ADH1B ADH1C ADH4 ADH6 AFM AGXT AGXT2 AKR1D1 AKR7A3 ALDH2 ALDH6A1 ALDOB ALPL ANG ANXA10 AOX1 APCS APOA5 APOF ARG1 ASS1 ATF5 ATOH8 AZGP1 BBOX1 BCHE BHMT C6 C7 C8A C8B C9 CA2 CAT CETP CFHR3 CFI CHST4 CLEC1B CLEC4G CLRN3 CMBL CNDP1 CPS1 CXCL2 CYP1A2 CYP2A6 CYP2C18 CYP2C8 CYP2C9 CYP2E1 CYP2J2 CYP39A1 CYP3A4 CYP4A11 CYP4V2 CYP8B1 DBH DCN DNASE1L3 DPT DPYS DUSP1 ECM1 EGR1 ENO3 EPHX2 F9 FBP1 FCN3 FETUB FMO3 FOS FOSB FTCD FXYD1 GADD45B GADD45G GBA3 GCGR GHR GLS2 GLYAT GNE GNMT GRHPR GSTA2 HAMP HAO1 HAO2 HBA2 HBB HGFAC HMGCL HP HPX HRG HSD17B13 HSD17B6 IGFALS IGFBP1 IGFBP3 INMT KBTBD11 KLKB1 KMO LCAT LEAP2 LECT2 LY6E MARCO MAT1A MBL2 MT1A MT1E MT1F MT1G MT1H MT1M MT1X MT2A MTTP NAT2 NDRG2 NNMT OGDHL OIT3 OTC PCK1 PDK4 PHGDH PLG PNPLA7 PON1 PON3 PPARGC1A PRODH2 PROZ PZP RBP4 RCL1 RDH16 RDH5 RND3 SAA4 SERPINA11 SHBG SLC10A1 SLC13A5 SLC19A3 SLC22A1 SLC27A2 SLC27A5 SLC38A2 SLC38A4 SLC39A5 SLCO1B3 SOCS2 SPP2 SRD5A2 ST3GAL6 SULT2A1 TACSTD2 TAT TDO2 THRSP TTC36 TTR UGT2B10 VIPR1 WDR72

**Notes.**

Abbreviations DEGs differentially expressed genes HCC hepatocellular carcinoma TCGA-LIHC the cancer genome atlas-liver hepatocellular carcinoma

### PPI and cluster analysis

**Figure 5:**
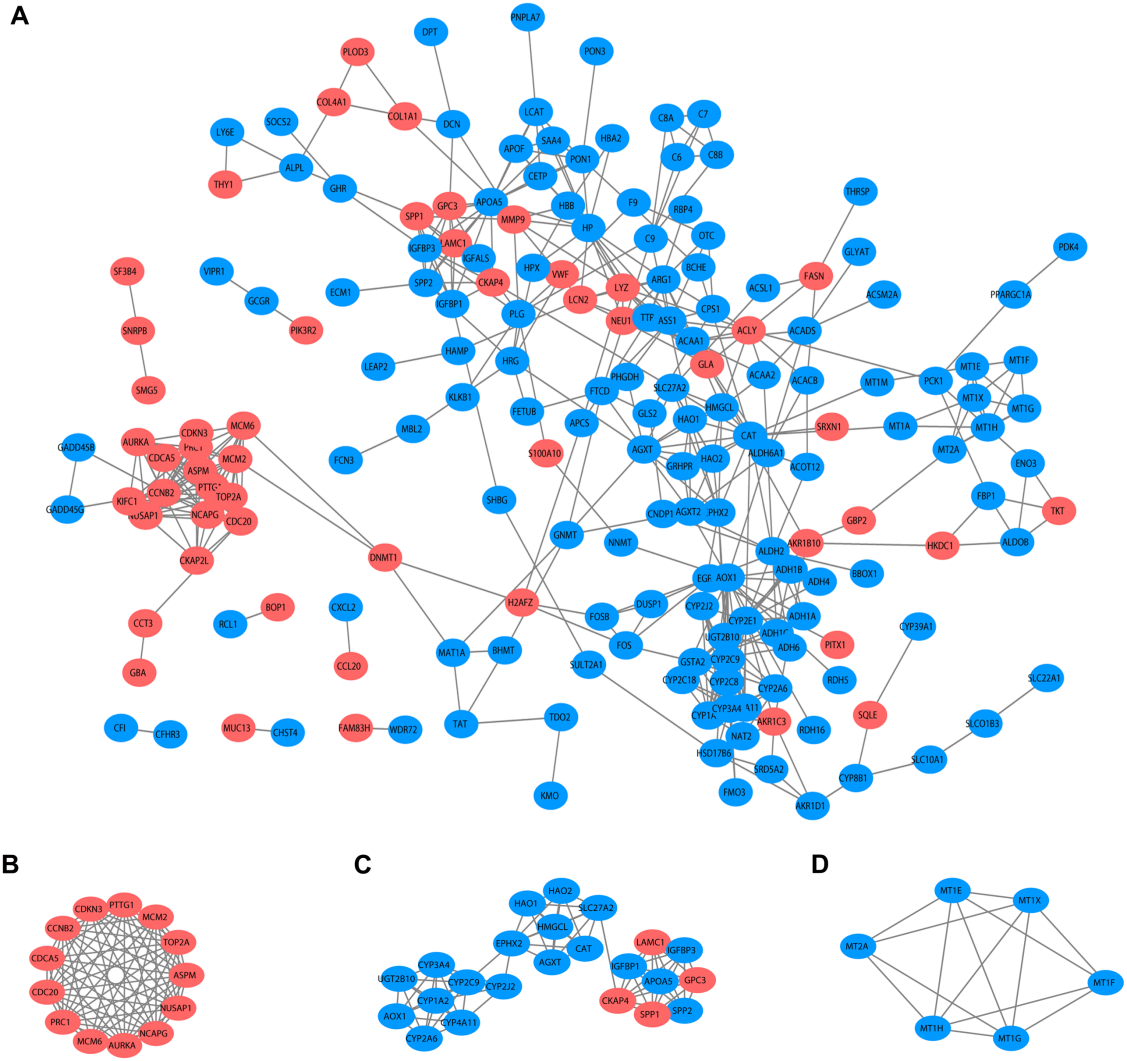
PPI network and clusters identification. (A) Interactions between 188 DEGs were detected through STRING with high confidence (combined score > 0.7). (B) Cluster 1 with 13 nodes and 72 edges. (C) Cluster 2 with 23 nodes and 77 edges. (D) Cluster 3 with 6 nodes and 14 edges. MCODE plugin was employed for the detection of clusters. The red nodes represent significantly upregulated genes, while blue nodes represent significantly downregulated genes. PPI, protein-protein interaction; DEGs, differentially expressed genes; MCODE, molecular complex detection.

Based on the published literature collected by STRING, 188 DEGs were filtered in the PPI network, and 486 edges are displayed in Fig. 5A. Furthermore, the MCODE plugin was applied to investigate highly interconnected regions, known as clusters, in the PPI network. The three clusters with the highest scores are shown in Figs. 5B, 5C and 5D, respectively. Cluster 1, consisting of 13 nodes and 72 edges, was highly enriched in cell division, nuclear division, cell cycle, chromosome segregation and condensation (Fig. 5B, Table S3). Twenty-three nodes and 77 edges were engaged in cluster 2 and were mainly associated with the epoxygenase P450 pathway and metabolic processes (Fig. 5C, Table S4). For Cluster 3, metallothioneins bind metals, and the response to metal ions and detoxification were significantly enriched (Fig. 5D, Table S5).

### Key gene selection and further analyses

**Figure 6:**
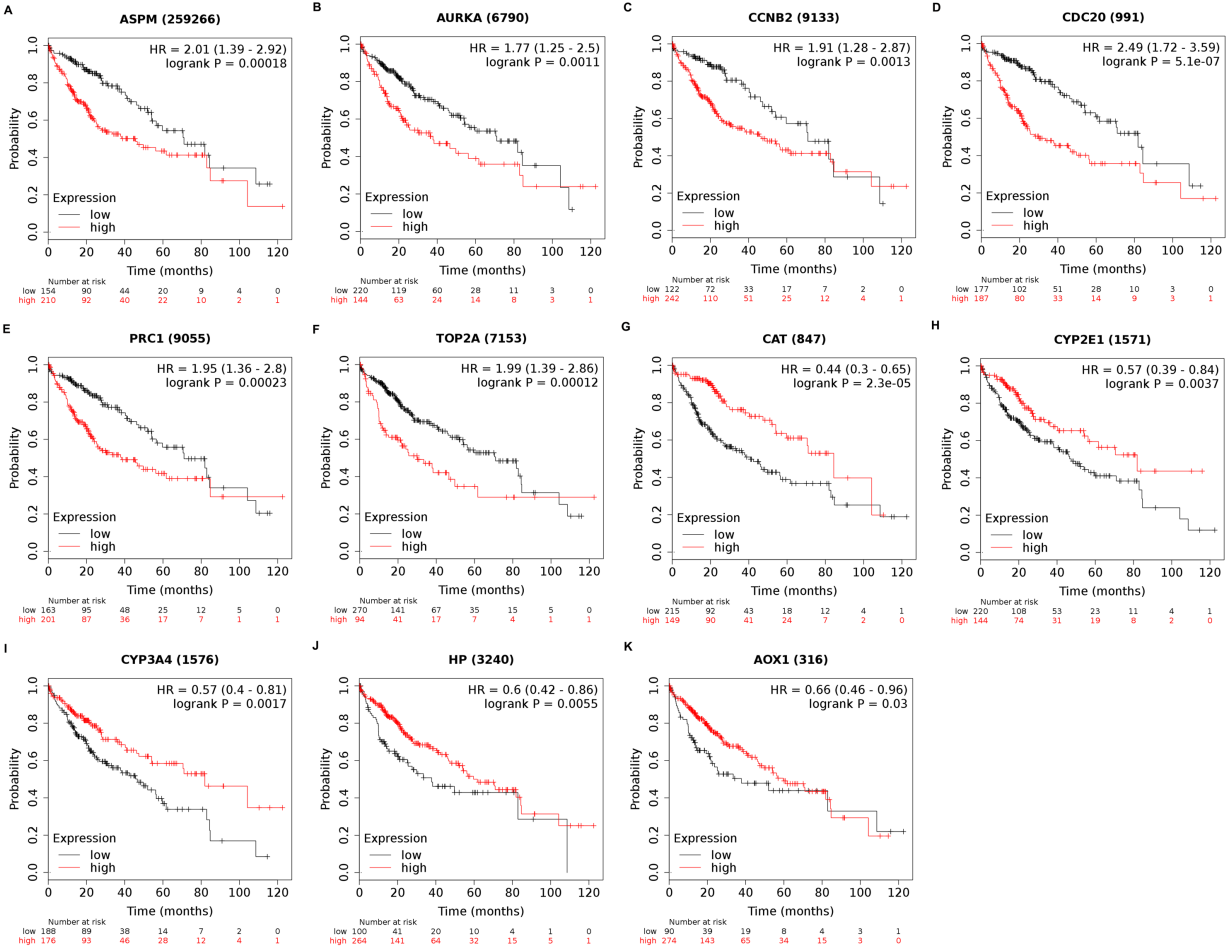
Overall survival analysis of 11 key genes in HCC patients. (A) ASPM; (B) AURKA; (C) CCNB2; (D) CDC20; (E) PRC1; (F) TOP2A; (G) CAT; (H) CYP2E1; (I) CYP3A4; (J) HP; (K) AOX1. *P*-value < 0.05 was considered statistically significant. In HCC samples, ASPM, AURKA, CCNB2, CDC20, PRC1 and TOP2A were upregulated while CAT, CYP2E1, CYP3A4, HP and AOX1 were downregulated. HCC, hepatocellular carcinoma; HR, hazard ratio.

Among the 188 nodes filtered in the PPI network, 30 hub genes had 10 or more interactions (degree ≥ 10), and the top 11 genes with the highest degree were AOX1, CYP2E1, HP, CCNB2, CDC20, ASPM, AURKA, CAT, CYP3A4, PRC1, and TOP2A (Table 4). These 11 key biomarkers were highly enriched in cell division, spindle organization and assembly, nuclear division and drug catabolic process (Table 5). Moreover, the OS of the key biomarkers was analyzed through the Kaplan–Meier Plotter database. The upregulation of ASPM, AURKA, CCNB2, CDC20, PRC1 or TOP2A in HCC patients indicated a grim outcome (Fig. 6).

**Table 2:** The top 10 (a ranking by *P*-value from Metascape) biological process of DEGs in HCC.

**Term**	**Description**	**Count**	**Log**_**10**_**(*P-*value)**
Upregulated			
GO:0000819	Sister chromatid segregation	9	−7.78
GO:0000070	Mitotic sister chromatid segregation	8	−7.31
GO:0051301	Cell division	13	−7.03
GO:0000280	Nuclear division	11	−6.90
GO:0098813	Nuclear chromosome segregation	9	−6.58
GO:0140014	Mitotic nuclear division	9	−6.54
GO:0048285	Organelle fission	11	−6.48
GO:0016137	Glycoside metabolic process	4	−6.26
GO:0007059	Chromosome segregation	9	−5.85
GO:0033044	Regulation of chromosome organization	9	−5.63
Downregulated			
GO:0032787	Monocarboxylic acid metabolic process	50	−35.48
GO:0071466	Cellular response to xenobiotic stimulus	26	−25.28
GO:0009410	Response to xenobiotic stimulus	30	−24.68
GO:0016054	Organic acid catabolic process	28	−22.88
GO:0046395	Carboxylic acid catabolic process	28	−22.88
GO:0044282	Small molecule catabolic process	33	−22.53
GO:1901615	Organic hydroxy compound metabolic process	35	−22.21
GO:0006805	Xenobiotic metabolic process	21	−21.89
GO:0008202	Steroid metabolic process	29	−21.88
GO:0006631	Fatty acid metabolic process	29	−20.04

**Notes.**

Abbreviations DEGs differentially expressed genes HCC hepatocellular carcinoma GO gene ontology

**Figure 7:**
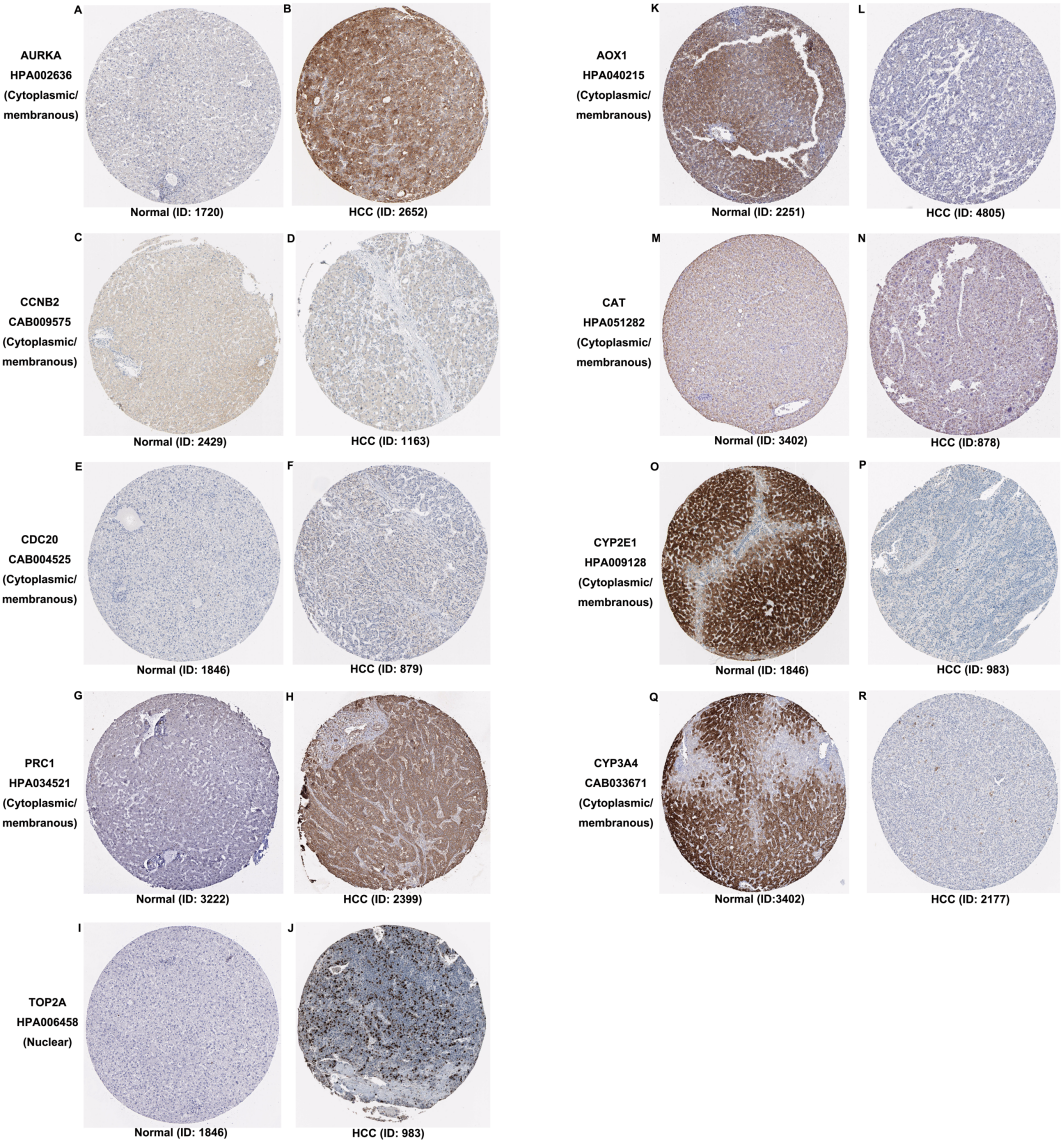
Expression and protein distribution of key genes in normal and HCC tissue measured by immunohistochemistry in the HPA database. (A–J) Expression and distribution of upregulated AURKA, CCNB2, CDC20, PRC1 or TOP2A proteins in normal and HCC tissues. (K–R) Expression and distribution of downregulated AOX1, CAT, CYP2E1, CYP3A4 proteins in normal and HCC tissues. Magnification, 100x. HCC, hepatocellular carcinoma; HPA, Human Protein Atlas.

Additionally, the expression and distribution of the key biomarkers in HCC samples and nontumor samples were measured through immunohistochemistry (Fig. 7). Unfortunately, immunohistochemistry results of ASPM and HP in HCC samples were not found in the HPA database. The antibodies used were as follows: AURKA (HPA002636), CCNB2 (CAB009575), CDC20 (CAB004525), PRC1 (HPA034521), TOP2A (HPA006458), AOX1 (HPA040215), CAT (HPA051282), CYP2E1 (HPA009128), and CYP3A4 (CAB033671). Upregulated AURKA (Figs. 7A, 7B), PRC1 (Figs. 7G, 7H) and TOP2A (Figs. 7I, 7J) were highly expressed in HCC tissue but undetectable or expressed at low levels in normal liver tissue. Downregulated AOX1 (Figs. 7K, 7L), CYP2E1 (Figs. 7O, 7P), and CYP3A4 (Figs. 7Q, 7R) were highly expressed in normal liver tissue but undetectable or expressed at low levels in HCC. The changes in CCNB2 (Figs. 7C, 7D), CDC20 (Figs. 7E, 7F), and CAT (Figs. 7M, 7N) between HCC tissue and normal tissue were not pronounced due to low abundance. Therefore, AURKA, PRC1, TOP2A, AOX1, CYP2E1, and CYP3A4 can be considered candidate liver-biopsy markers for high risk of developing HCC and poor prognosis in HCC.

### Prognostic miRNA identification and survival analysis

**Figure 8:**
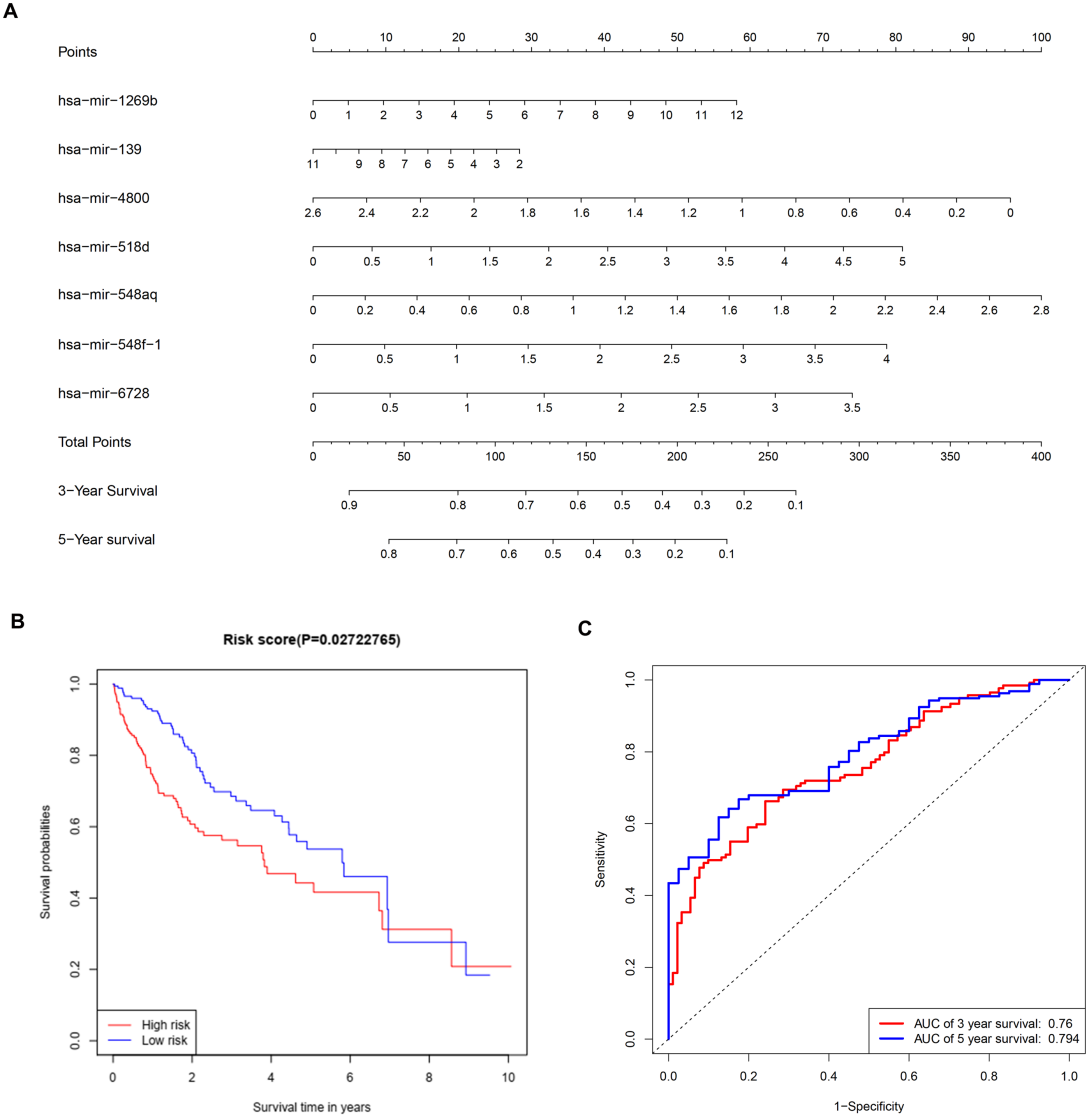
Construction of a prognostic system for HCC based on seven microRNAs (hsa-mir-1269b, hsa-mir-518d, hsa-mir-548aq, hsa-mir-548f-1, hsa-mir-6728, hsa-mir-139 and hsa-mir-4800). (A) The nomogram predicting overall survival rates of HCC patients based on seven microRNAs. (B) Survival curves of patients with low risk and high risk. The cut-off score of low and high risk was 0.936. (C) ROC curves in 3 and 5 years with an AUC value of 0.76 and 0.794, respectively. HCC, hepatocellular carcinoma.

To further investigate the connections between miRNAs and gene expression, prognostic analysis was also performed in miRNAs. A total of 409 miRNAs were identified as differentially expressed miRNAs (Table S6) and were included in univariate analysis. Forty-four miRNAs with a *P*-value < 0.1 in univariate analysis (Table S7) were selected as hits and were enrolled in further multivariate Cox regression analysis. Finally, seven miRNAs (hsa-mir-1269b, hsa-mir-139, hsa-mir-4800, hsa-mir-518d, hsa-mir-548aq, hsa-mir-548f-1, hsa-mir-6728) were identified as prognostic factors (*P*-value < 0.05) impacting the survival of HCC patients (Table 6). Based on the seven miRNAs, a prognostic nomogram predicting 3-year and 5-year survival is shown in Fig. 8A. The cut-off score of low and high risk was 0.936 (Table S8). Meanwhile, the survival curves of patients with low risk and high risk, and the time-dependent ROC curves at 3 years (AUC = 0.76) and 5 years (AUC = 0.794) are plotted in Figs. 8B and 8C, respectively. Moreover, according to miRWalk, most key genes identified were potential target genes (score > 0.8) of these prognostic miRNAs (Table 6).

**Table 3:** The top 10 (a ranking by *P*-value from Metascape) significantly enriched pathways of DEGs in HCC.

**Term**	**Description**	**Count**	**Log**_**10**_**(*P-*value)**
Upregulated			
R-HSA-1474244	Extracellular matrix organization	8	−5.11
hsa04512	ECM-receptor interaction	5	−5.08
R-HSA-69278	Cell Cycle, Mitotic	10	−4.90
R-HSA-1474290	Collagen formation	5	−4.88
hsa05146	Amoebiasis	5	−4.74
R-HSA-2243919	Crosslinking of collagen fibrils	3	−4.54
R-HSA-2022090	Assembly of collagen fibrils and other multimeric structures	4	−4.28
hsa04510	Focal adhesion	6	−4.25
R-HSA-1640170	Cell Cycle	10	−4.24
hsa04110	Cell cycle	5	−4.21
Downregulated			
R-HSA-211859	Biological oxidations	29	−26.90
R-HSA-211945	Phase I - Functionalization of compounds	20	−21.93
hsa00830	Retinol metabolism	17	−21.33
hsa05204	Chemical carcinogenesis	16	−17.86
R-HSA-556833	Metabolism of lipids	35	−17.72
hsa00982	Drug metabolism - cytochrome P450	15	−17.43
R-HSA-8978868	Fatty acid metabolism	19	−16.05
hsa00980	Metabolism of xenobiotics by cytochrome P450	14	−15.49
R-HSA-5661231	Metallothioneins bind metals	8	−14.86
R-HSA-71291	Metabolism of amino acids and derivatives	23	−14.06

**Notes.**

Abbreviations DEGs differentially expressed genes HCC hepatocellular carcinoma

**Table 4:** Thirty hub genes in the PPI network constructed by STRING (degree ≥ 10).

**Gene names**	**Description**	**Alteration**	**Count**
AOX1	Aldehyde oxidase 1	Down	19
CYP2E1	Cytochrome P450 family 2 subfamily E member 1	Down	19
HP	Haptoglobin	Down	16
CCNB2	Cyclin B2	Up	15
CDC20	Cell division cycle 20	Up	15
ASPM	Abnormal spindle microtubule assembly	Up	14
AURKA	Aurora kinase A	Up	14
CAT	Catalase	Down	14
CYP3A4	Cytochrome P450 family 3 subfamily A member 4	Down	14
PRC1	Protein regulator of cytokinesis 1	Up	14
TOP2A	DNA topoisomerase II alpha	Up	14
ALDH2	Aldehyde dehydrogenase 2 family member	Down	13
APOA5	Apolipoprotein A5	Down	13
CYP1A2	Cytochrome P450 family 1 subfamily A member 2	Down	13
NCAPG	Non-SMC condensin I complex subunit G	Up	13
AGXT	Alanine-glyoxylate and serine-pyruvate aminotransferase	Down	12
CYP2C9	Cytochrome P450 family 2 subfamily C member 9	Down	12
NUSAP1	Nucleolar and spindle associated protein 1	Up	12
PTTG1	PTTG1 regulator of sister chromatid separation, securin	Up	12
ACAA1	Acetyl-CoA acyltransferase 1	Down	11
GSTA2	Glutathione S-transferase alpha 2	Down	11
LYZ	Lysozyme	Down	11
MCM2	Minichromosome maintenance complex component 2	Up	11
UGT2B10	UDP glucuronosyltransferase family 2 member B10	Down	11
ACLY	ATP citrate lyase	Up	10
ARG1	Arginase 1	Down	10
CYP2C8	Cytochrome P450 family 2 subfamily C member 8	Down	10
CYP4A11	Cytochrome P450 family 4 subfamily A member 11	Down	10
IGFBP3	Insulin like growth factor binding protein 3	Down	10
MCM6	Minichromosome maintenance complex component 6	Up	10

**Notes.**

Abbreviations PPI protein-protein interaction

**Table 5:** Top 20 (a ranking by *P*-value from Metascape) enriched functions and pathways of key biomarkers.

**Term**	**Description**	**Count**	**Log**_**10**_**(*P-*value)**
GO:0051301	Cell division	7	−8.78
GO:0042737	Drug catabolic process	4	−6.44
GO:0000280	Nuclear division	5	−6.23
GO:0000922	Spindle pole	4	−6.19
GO:0051302	Regulation of cell division	4	−6.14
GO:0140013	Meiotic nuclear division	4	−6.10
GO:0048285	Organelle fission	5	−6.02
GO:0007051	Spindle organization	4	−5.96
GO:1903046	Meiotic cell cycle process	4	−5.95
GO:0051321	Meiotic cell cycle	4	−5.46
hsa00982	Drug metabolism - cytochrome P450	3	−5.42
GO:0005815	Microtubule organizing center	5	−4.99
GO:0005819	Spindle	4	−4.89
GO:0007052	Mitotic spindle organization	3	−4.84
hsa04114	Oocyte meiosis	3	−4.67
GO:0051225	Spindle assembly	3	−4.66
GO:1902850	Microtubule cytoskeleton organization involved in mitosis	3	−4.57
GO:0020037	Heme binding	3	−4.56
GO:0007292	Female gamete generation	3	−4.55
GO:0046906	Tetrapyrrole binding	3	−4.47

**Notes.**

Abbreviations GO gene ontology

## Discussion

In the present study, 262 aberrantly expressed genes in HCC samples were disclosed by integrated bioinformatics analyses. The functional and signaling pathway enrichment analysis indicated the underlying mechanisms in HCC development. Among these genes, 11 key genes were identified through PPI analysis and their prognostic value in HCC patients was validated by OS analysis and immunohistochemistry. Meanwhile, univariate and multivariate Cox regression analysis revealed the prognostic application of seven miRNAs. Interestingly, according to miRWalk, most key genes were potentially regulated by these miRNAs.

Genomic, epigenetic and transcriptional alterations, as well as subsequent multistep processes, play crucial roles in the occurrence, development, metastasis and prognosis of HCC (Dhanasekaran et al., 2019; Llovet et al., 2018). Combined with public biological databases (e.g., GO and KEGG), the development of high-throughput detection technology provides us with an opportunity to systematically explore “interesting” gene lists on a genome-wide scale and sort out correlative biological processes (Shan, Chen & Jia, 2019; Shen et al., 2019). To identify reproducible and robust genetic alterations, integrated analyses based on multiple datasets gradually replace single cohort analyses. Thus, in the present study, we recruited four datasets from GEO and one dataset from TCGA, including 789 HCC samples and 384 nontumor samples in total, to perform a meta-analysis and identify consensus alterations. Notably, all data from GEO in our study were generated by Illumina expression beadchip platforms, and our study was the first large-scale integrated bioinformatics analysis of the data from Illumina BeadArray platforms and TCGA database.

According to the functional, signaling pathway and protein-protein interaction analyses, DEGs had noticeable impacts on multiple cellular activities, including DNA structure, cell division, metabolism, and microtubule cytoskeleton organization. Three clusters identified in PPI network mainly functioned in cell division and metabolic processes. These findings were consistent with our knowledge (Jiang et al., 2015; Kastan & Bartek, 2004; Liu et al., 2016; Loong & Yeo, 2014; Shoieb et al., 2019). Among 30 hub genes, upregulated ASPM, AURKA, CCNB2, CDC20, PRC1 and TOP2A and downregulated AOX1, CAT, CYP2E1, CYP3A4 and HP, were considered key biomarkers in HCC. Overall survival analysis indicated that the aberrant expression of these genes correlated with a poor prognosis. With the aid of immunohistochemistry, AURKA, PRC1, TOP2A, AOX1, CYP2E1, and CYP3A4 were considered candidate liver-biopsy markers for HCC. According to the instruction on HPA database, all images were manually annotated by a specialist followed by verification by a second specialist using fixed guidelines. Basic annotation parameters include an evaluation of staining intensity (negative, weak, moderate or strong), fraction of stained cells (<25%, 25–75% or >75%) and subcellular localization (nuclear and/or cytoplasmic/membranous). Thus, they were deemed reliable because of the homogeneous processes and fixing guidelines, although all immunohistochemistry images were manually annotated. However, the significance of these proteins in HCC requires further validation due to the limited number of samples in HPA, unpaired comparison and lack of statistical analysis.

The overexpression of ASPM, which contributes to neurogenesis and cell proliferation, is an independent risk factor for early tumor recurrence regardless of p53 mutation status and poor prognosis of HCC (Lin et al., 2008). TOP2A, which is a mitotic gene and highly expressed at the G2/M phase, correlates with early age onset, shorter patient survival and chemoresistance (Wong et al., 2009). TRRAP/KAT5, which activates TOP2A, has been reported to inhibit HCC cell growth through induction of p53-independent and p21-independent senescence (Kwan et al., 2020). CCNB2, CDC20 and PRC1 are the three most commonly reported upregulated genes in HCC through bioinformatics analyses (Chen et al., 2016a; Gao et al., 2018; Li et al., 2014; Liu et al., 2018; Wang et al., 2019b). CCNB2, as a component of the cell cycle regulatory machinery, is associated with the Golgi region (Draviam et al., 2001; Jackman, Firth & Pines, 1995) and plays an important role in regulating the G2/M transition (Gui & Homer, 2013; Li et al., 2018a). CDC20 is essential to chromosome segregation and mitotic exit and regulates the cell cycle at multiple time points (Kapanidou, Curtis & Bolanos-Garcia, 2017). PRC1 exerts an impact on the formation of microtubule architectures and subsequent cell shape formation and cytokinesis regulation and promotes early recurrence of HCC associated with the Wnt/ β-catenin signaling pathway (Chen et al., 2016a). AURKA promotes cancer metastasis in HCC by inducing epithelial-mesenchymal transition and cancer stem cell behaviors through the PI3K/AKT pathway (Chen et al., 2017). Alisertib, an AURKA inhibitor, has been demonstrated to potently inhibit cell viability and induce apoptosis in HCC cells (Li et al., 2018b). The elevated expression levels of PRC1, TOP2A, and AURKA in HCC tissue were also confirmed by immunohistochemistry and appear to be candidate biopsy markers for high risk of developing HCC and poor prognosis in HCC patients.

It has been reported that AOX1 interacts with ABCA1 to regulate ABCA1-related cellular functions such as lipid efflux and phagocytosis in hepatocytes and is highly expressed in normal human liver tissue (Sigruener et al., 2007). In contrast, reduced expression of AOX1 is detected in HCC cells and is highly correlated with higher tumor stage, distant metastases or positive lymph node status (Sigruener et al., 2007). Haptoglobin (HP) is an acidic glycoprotein tetramer that is mainly secreted in the liver. Its expression is low during the active proliferation of progenitor cells but increases when the cells reach confluency (Guillouzo et al., 2007). CAT, a well-described oxidative stress biomarker, plays an important role in inflammation and the prevention of apoptosis and serves as a growth promoting factor in a wide spectrum of tissues (Goyal & Basak, 2010; Miyamoto et al., 1996). Hence, it is reasonable for CAT to be a key biomarker in hepatocarcinogenesis and development. Both CYP2E1 and CYP3A4, as members of the cytochrome P450 family which contributes to drug metabolism, lipid synthesis and homeostasis (Manikandan & Nagini, 2018), have been reported to be less expressed in HCC (Chen et al., 2014; Hu et al., 2019).

**Table 6:** Identification of prognostic microRNAs in HCC and target genes predicted by miRWalk (score > 0.8).

MicroRNA	Alteration	Univariate		Multivariate		Predicted target genes
		**HR**	***P*-value**	**HR**	***P*-value**	
hsa-mir-1269b	Up	1.0550	0.0747	1.0012	0.0000	ASPM AURKA CAT CCNB2 CDC20 CYP2E1 PRC1 TOP2A
hsa-mir-139	Down	0.9251	0.0669	0.9976	0.0028	AURKA CAT CDC20 CCNB2 CYP2E1 HP PRC1 TOP2A
hsa-mir-4800	Down	0.7442	0.0525	0.7343	0.0038	ASPM AURKA CAT CCNB2 CDC20 CYP2E1 CYP3A4 HP PRC1 TOP2A
hsa-mir-518d	Up	1.2729	0.0401	1.1077	0.0026	ASPM AURKA CDC20 CCNB2 CYP2E1 CYP3A4 HP PRC1 TOP2A
hsa-mir-548aq	Up	1.7916	0.0020	1.3604	0.0150	ASPM
hsa-mir-548f-1	Up	1.2938	0.0307	1.1711	0.0111	
hsa-mir-6728	Up	1.4644	0.0010	1.1565	0.0381	ASPM AURKA CAT CDC20 CYP2E1 CYP3A4 HP PRC1 TOP2A

**Notes.**

Abbreviations HCC hepatocellular carcinoma GO gene ontology

Multivariate Cox regression analysis offered a prognostic model involving seven miRNAs. By reviewing the published literature, we found that hsa-miR-1269b and hsa-miR-139 had been reported to be dysregulated in HCC. Hsa-miR-1269b promotes malignancy in HCC by upregulating cell division cycle 40 homolog (Kong et al., 2016). Downregulation of hsa-miR-139 is associated with poor outcome (Wang et al., 2019a) and the long noncoding RNA SNHG3/miR-139-5p/BMI1 axis may be one of the potential signaling pathways (Wu et al., 2019). However, the correlative mechanisms and pathways of hsa-miR-4800, hsa-miR-518d, hsa-mir-548aq, hsa-mir-548f-1 and hsa-mir-6728 in HCC have not been determined. By executing the TarPmiR algorithm for target site prediction in miRWalk, most key genes were considered candidate target genes of the indicated miRNAs and the same mRNA was likely to be controlled by various miRNAs, which is a common phenomenon. Currently, the interactions between these molecules lack solid supports, and experimental evidence is required to elucidate the underlying mechanisms.

The emergence and advances in the bioinformatics field accelerate the development of biology. Bioinformatics tools provide opportunities for handling big data that are impossible to manage manually. However, the heterogeneity of how data are generated, assembled, annotated and displayed will substantially affect the results. It is a common limitation of bioinformatics analyses. Meanwhile, the clinical features of the patients such as HBV/HCV infection, age, alcohol consumption and cancer stages were not analysed in our study. These factors may have important impacts on gene and miRNA expression. The correlation between biomarkers and cancer stages also requires further research to provide useful references and insights into research and the clinic. Moreover, in-depth research should be conducted with the combination of traditional biology technologies to validate the findings from bioinformatics analyses, particularly the interactions between miRNAs and potential target mRNAs.

## Conclusions

The present study systematically analyzed multiple transcriptome profiles and summarized a list of differentially expressed genes and miRNAs in HCC. To the best of our knowledge, no such large-scale investigation on Illumina BeadArray platforms was performed previously. Eleven key genes and a 7-miRNA model provide biomarkers for the surveillance and prognosis of HCC. This study decoded the alterations in HCC at the molecular and functional levels and offered potential targets for therapy.

##  Supplemental Information

10.7717/peerj.8930/supp-1 Table S1 DEGs identified in HCC samples from GSE36376, GSE39791, GSE57957, and GSE87630

10.7717/peerj.8930/supp-2 Table S2 DEGs identified in HCC samples in TCGA

10.7717/peerj.8930/supp-3 Table S3 The top 20 (a ranking by *P*-value from Metascape) enriched GO and pathways of genes in cluster 1

10.7717/peerj.8930/supp-4 Table S4 The top 20 (a ranking by *P*-value from Metascape) enriched GO and pathways of genes in cluster 2

10.7717/peerj.8930/supp-5 Table S5 The top 20 (a ranking by *P*-value from Metascape) enriched GO and pathways of genes in cluster 3

10.7717/peerj.8930/supp-6 Table S6 Differentially expressed microRNAs in HCC samples in TCGA

10.7717/peerj.8930/supp-7 Table S7 Univariate analysis of microRNAs in HCC (*P*-value < 0.1)

10.7717/peerj.8930/supp-8 Table S8 Risk scores of HCC samples based on the microRNA prognostic system

10.7717/peerj.8930/supp-9 Supplemental Information 1 R scripts

## Additional Information and Declarations

### Data Availability

The following information was supplied regarding data availability: All the raw data are available at GEO: GSE36376, GSE39791, GSE57957, and GSE87630 and at TCGA (search term: LIHC). The code files are available in a Supplemental File.
