# Supplementary material for: Transcriptome analysis revealed key prognostic genes and microRNAs in hepatocellular carcinoma
Source: PeerJ. 2020 Apr 8;8:e8930. doi: 10.7717/peerj.8930 (PMC7150540; doi:10.7717/peerj.8930)
Supplement: Table S1 [file peerj-08-8930-s001.docx]

| **DEGs** | **Gene names** |
| --- | --- |
| **Upregulated** | ACLY ACTA2 ACTG2 AKR1B10 AKR1C3 ARMET ASPM ATP6AP1 AURKA BAIAP2L2 BOP1 BRSK1 C1orf85 CAP2 CCL20 CCNB2 CCT3 CD24 CDC20 CDCA5 CDKN3 CKAP2L CKAP4 CLIC1 CNIH4 COL1A1 COL4A1 COL5A2 COPA COX7B2 CPD CSTB CTSA DNAJB11 DNMT1 EEF1A2 EPRS FADS1 FAM83H FASN GARS GBA GBP2 GLA GPAA1 GPC3 GPR172A H2AFZ HGS HKDC1 HSP90AB1 HSPB1 IGF2BP2 ILF2 IRAK1 IRX3 KIFC1 LAMC1 LAPTM4B LCN2 LMNA LOC651816 LOC728492 LOC731049 LOXL4 LSM4 LYZ MCM2 MCM6 MMP9 MUC13 NCAPG NCSTN NDUFA4L2 NEU1 NSMCE2 NUSAP1 PAFAH1B3 PARP1 PEA15 PIK3R2 PITX1 PLOD3 PLVAP PRC1 PSMB4 PTTG1 RAP2A RPL15 RRAGD S100A10 S100P SAC3D1 SAE1 SF3B4 SIPA1L2 SLC25A39 SLC39A1 SMG5 SNRPB SPINK1 SPP1 SQLE SRXN1 STIP1 TEAD2 TFRC THY1 TKT TMEM106C TMEM45B TOMM40 TOP2A TUBA1B TUBA1C UBD VWF |
| **Downregulated** | A2M ACAA1 ACAA2 ACACB ACAD11 ACADS ACOT12 ACSL1 ACSM2A ADAMTSL2 ADH1A ADH1B ADH1C ADH4 ADH6 AFM AGXT AGXT2 AKR1D1 AKR7A3 ALDH1L1 ALDH2 ALDH6A1 ALDOB ALPL ANG ANXA10 AOX1 APCS APOA5 APOC4 APOF AQP9 ARG1 ASS1 ATF5 ATOH8 AXUD1 AZGP1 BBOX1 BCHE BHMT C10orf116 C10orf65 C14orf68 C20orf127 C4BPA C6 C7 C8A C8B C8orf4 C9 C9orf103 CA2 CAT CETP CFHR3 CFI CHST4 CIDEB CLEC1B CLEC4G CLRN3 CMBL CNDP1 CPS1 CXCL2 CYP1A2 CYP2A6 CYP2A7 CYP2C18 CYP2C8 CYP2C9 CYP2E1 CYP2J2 CYP39A1 CYP3A4 CYP4A11 CYP4F12 CYP4V2 CYP8B1 CYR61 DAK DBH DCN DCXR DEFB1 DNASE1L3 DPT DPYS DUSP1 ECM1 EGR1 ENO3 EPHX2 EVC2 F12 F9 FBP1 FCN3 FETUB FIS FLJ21986 FMO3 FOS FOSB FTCD FXYD1 G6PC GADD45B GADD45G GBA3 GCGR GHR GLS2 GLYAT GNE GNMT GPT GPT2 GRHPR GSTA2 GSTA5 HAMP HAO1 HAO2 HBA2 HBB HGFAC HMGCL HP HPD HPX HRG HRSP12 HSD11B1 HSD17B13 HSD17B2 HSD17B6 IGFALS IGFBP1 IGFBP3 INMT JMJD5 KBTBD11 KLKB1 KMO LCAT LEAP2 LECT2 LOC284422 LOC388503 LOC441019 LOC554235 LOC642113 LOC652493 LOC653498 LOC728811 LY6E MARCO MAT1A MBL2 MT1A MT1E MT1F MT1G MT1H MT1M MT1X MT2A MTE MTTP NAT2 NDRG2 NNMT OGDHL OIT3 OTC PCDH24 PCK1 PDK4 PHGDH PIPOX PLG PNPLA7 PON1 PON3 PPARGC1A PRODH2 PROZ PZP RBP4 RCL1 RDH16 RDH5 RND3 SAA4 SDS SERPINA11 SHBG SLC10A1 SLC13A5 SLC19A3 SLC22A1 SLC27A2 SLC27A5 SLC38A2 SLC38A4 SLC39A5 SLCO1B3 SNORD13 SOCS2 SPP2 SRD5A2 ST3GAL6 SULT2A1 TACSTD2 TAT TDO2 THRSP TMEM27 TTC36 TTR UGT2B10 VIPR1 WDR72 |

Abbreviations: DEGs, differentially expressed genes; HCC, hepatocellular carcinoma.
