# Supplementary material for: Transcriptome analysis revealed key prognostic genes and microRNAs in hepatocellular carcinoma
Source: PeerJ. 2020 Apr 8;8:e8930. doi: 10.7717/peerj.8930 (PMC7150540; doi:10.7717/peerj.8930)
Supplement: Table S3 [file peerj-08-8930-s003.docx]

| **Term** | **Description** | **Count** | **Log_10_(*P-*value)** |
| --- | --- | --- | --- |
| GO:0051301 | Cell division | 10 | -13.67 |
| GO:0000280 | Nuclear division | 9 | -13.13 |
| GO:0048285 | Organelle fission | 9 | -12.75 |
| R-HSA-69278 | Cell Cycle, Mitotic | 9 | -12.08 |
| GO:0000819 | Sister chromatid segregation | 7 | -11.53 |
| R-HSA-1640170 | Cell Cycle | 9 | -11.38 |
| GO:0098813 | Nuclear chromosome segregation | 7 | -10.54 |
| GO:0140014 | Mitotic nuclear division | 7 | -10.51 |
| GO:0000070 | Mitotic sister chromatid segregation | 6 | -10.00 |
| GO:0007059 | Chromosome segregation | 7 | -9.93 |
| hsa04110 | Cell cycle | 5 | -8.39 |
| GO:0044772 | Mitotic cell cycle phase transition | 7 | -8.13 |
| GO:0030261 | Chromosome condensation | 4 | -8.05 |
| GO:0071103 | DNA conformation change | 6 | -8.03 |
| GO:0044770 | Cell cycle phase transition | 7 | -7.92 |
| GO:0007088 | Regulation of mitotic nuclear division | 5 | -7.75 |
| GO:0140013 | Meiotic nuclear division | 5 | -7.67 |
| GO:1903046 | Meiotic cell cycle process | 5 | -7.48 |
| GO:0051783 | Regulation of nuclear division | 5 | -7.46 |
| GO:0006323 | DNA packaging | 5 | -7.24 |

Abbreviations: GO, gene ontology.
