# Supplementary material for: Transcriptome analysis revealed key prognostic genes and microRNAs in hepatocellular carcinoma
Source: PeerJ. 2020 Apr 8;8:e8930. doi: 10.7717/peerj.8930 (PMC7150540; doi:10.7717/peerj.8930)
Supplement: Table S4 [file peerj-08-8930-s004.docx]

| **Term** | **Description** | **Count** | **Log_10_(*P-*value)** |
| --- | --- | --- | --- |
| GO:0019373 | Epoxygenase P450 pathway | 6 | -13.85 |
| GO:0006631 | Fatty acid metabolic process | 11 | -13.78 |
| R-HSA-8957275 | Post-translational protein phosphorylation | 8 | -13.25 |
| GO:0001676 | Long-chain fatty acid metabolic process | 8 | -13.18 |
| R-HSA-9033241 | Peroxisomal protein import | 7 | -12.86 |
| GO:0032787 | Monocarboxylic acid metabolic process | 12 | -12.85 |
| hsa00830 | Retinol metabolism | 7 | -12.76 |
| R-HSA-381426 | Regulation of Insulin-like Growth Factor transport and uptake by Insulin-like Growth Factor Bi | 8 | -12.73 |
| GO:0006625 | Protein targeting to peroxisome | 7 | -12.62 |
| GO:0072662 | Protein localization to peroxisome | 7 | -12.62 |
| GO:0072663 | Establishment of protein localization to peroxisome | 7 | -12.62 |
| GO:0043574 | Peroxisomal transport | 7 | -12.57 |
| GO:0007031 | Peroxisome organization | 7 | -12.07 |
| hsa04146 | Peroxisome | 7 | -11.99 |
| GO:0019369 | Arachidonic acid metabolic process | 6 | -11.14 |
| GO:0008202 | Steroid metabolic process | 9 | -10.98 |
| GO:0042738 | Exogenous drug catabolic process | 5 | -10.69 |
| R-HSA-211981 | Xenobiotics | 5 | -10.59 |
| R-HSA-211897 | Cytochrome P450 - arranged by substrate type | 6 | -10.49 |
| R-HSA-2142670 | Synthesis of epoxy and dihydroxyeicosatrienoic acids | 4 | -10.36 |

Abbreviations: GO, gene ontology.
