# Supplementary material for: Transcriptome analysis revealed key prognostic genes and microRNAs in hepatocellular carcinoma
Source: PeerJ. 2020 Apr 8;8:e8930. doi: 10.7717/peerj.8930 (PMC7150540; doi:10.7717/peerj.8930)
Supplement: Table S5 [file peerj-08-8930-s005.docx]

| **Term** | **Description** | **Count** | **Log_10_(*P-*value)** |
| --- | --- | --- | --- |
| R-HSA-5661231 | Metallothioneins bind metals | 6 | -20.78 |
| R-HSA-5660526 | Response to metal ions | 6 | -19.96 |
| GO:0010273 | Detoxification of copper ion | 6 | -19.74 |
| GO:1990169 | Stress response to copper ion | 6 | -19.74 |
| GO:0061687 | Detoxification of inorganic compound | 6 | -19.35 |
| GO:0097501 | Stress response to metal ion | 6 | -19.35 |
| GO:0071294 | Cellular response to zinc ion | 6 | -18.44 |
| GO:0071280 | Cellular response to copper ion | 6 | -17.76 |
| GO:0006882 | Cellular zinc ion homeostasis | 6 | -17.15 |
| GO:0071276 | Cellular response to cadmium ion | 6 | -17.07 |
| GO:0055069 | Zinc ion homeostasis | 6 | -17.00 |
| GO:0046688 | Response to copper ion | 6 | -16.59 |
| hsa04978 | Mineral absorption | 6 | -16.18 |
| GO:0010043 | Response to zinc ion | 6 | -16.08 |
| GO:0046686 | Response to cadmium ion | 6 | -15.61 |
| GO:0046916 | Cellular transition metal ion homeostasis | 6 | -14.16 |
| GO:0055076 | Transition metal ion homeostasis | 6 | -13.64 |
| GO:0098754 | Detoxification | 6 | -13.64 |
| GO:0071248 | Cellular response to metal ion | 6 | -12.66 |
| GO:0071241 | Cellular response to inorganic substance | 6 | -12.31 |

Abbreviations: GO, gene ontology.
