# Supplementary material for: Transcriptome analysis revealed key prognostic genes and microRNAs in hepatocellular carcinoma
Source: PeerJ. 2020 Apr 8;8:e8930. doi: 10.7717/peerj.8930 (PMC7150540; doi:10.7717/peerj.8930)
Supplement: Table S7 [file peerj-08-8930-s007.docx]

| MicroRNA | Univariate | | MicroRNA | Univariate | |
| --- | --- | --- | --- | --- | --- |
|  | **HR** | ***P*-value** |  | **HR** | ***P*-value** |
| hsa-mir-4771-2 | 1.7238 | 0.0004 | hsa-mir-5589 | 0.8980 | 0.0438 |
| hsa-mir-4778 | 1.8279 | 0.0007 | hsa-mir-5584 | 1.3701 | 0.0461 |
| hsa-mir-6728 | 1.4644 | 0.0010 | hsa-mir-551a | 1.2168 | 0.0469 |
| hsa-mir-548aq | 1.7916 | 0.0020 | hsa-mir-3125 | 1.3722 | 0.0504 |
| hsa-mir-7156 | 1.3505 | 0.0021 | hsa-mir-490 | 0.8580 | 0.0521 |
| hsa-mir-3680-1 | 1.2972 | 0.0027 | hsa-mir-4800 | 0.7442 | 0.0525 |
| hsa-mir-147b | 1.2296 | 0.0038 | hsa-mir-3189 | 1.1517 | 0.0546 |
| hsa-mir-2682 | 1.6474 | 0.0046 | hsa-mir-1270 | 1.1531 | 0.0549 |
| hsa-mir-3660 | 1.3657 | 0.0060 | hsa-mir-6735 | 1.2483 | 0.0558 |
| hsa-mir-3911 | 1.4475 | 0.0067 | hsa-mir-3140 | 1.1840 | 0.0567 |
| hsa-mir-105-1 | 1.0993 | 0.0081 | hsa-mir-1257 | 1.4061 | 0.0662 |
| hsa-mir-105-2 | 1.0948 | 0.0096 | hsa-mir-139 | 0.9251 | 0.0669 |
| hsa-mir-7-3 | 1.2107 | 0.0139 | hsa-mir-4427 | 0.5979 | 0.0696 |
| hsa-mir-137 | 1.1847 | 0.0140 | hsa-mir-1269b | 1.0550 | 0.0747 |
| hsa-mir-767 | 1.0928 | 0.0249 | hsa-mir-4502 | 1.4449 | 0.0822 |
| hsa-mir-5706 | 1.1565 | 0.0255 | hsa-mir-373 | 1.0918 | 0.0839 |
| hsa-mir-561 | 1.1630 | 0.0283 | hsa-mir-4741 | 1.1887 | 0.0840 |
| hsa-mir-548f-1 | 1.2938 | 0.0307 | hsa-mir-9-1 | 1.0647 | 0.0845 |
| hsa-mir-5003 | 1.1879 | 0.0322 | hsa-mir-3689a | 1.3378 | 0.0876 |
| hsa-mir-892c | 1.4091 | 0.0323 | hsa-mir-4658 | 1.1882 | 0.0892 |
| hsa-mir-1915 | 1.2307 | 0.0373 | hsa-mir-19a | 0.9335 | 0.0929 |
| hsa-mir-518d | 1.2729 | 0.0401 | hsa-mir-548a-3 | 1.3063 | 0.0968 |

Abbreviations: HCC, hepatocellular carcinoma.
